# Heterogeneity and Clinical Translation of Cardiac-Derived Extracellular Vesicles in Heart Failure: From Mechanisms to Precision Therapeutics

**DOI:** 10.31083/RCM46117

**Published:** 2026-01-23

**Authors:** Xiao-Lin Li, Zhen-Xun Wan, Hang Jiang, Yang Luo, Ming-Tai Chen, Han-Yu Hu, Qiu-Yu Liu, Gang Luo, Meng-Nan Liu

**Affiliations:** ^1^Department of Cardiovascular Disease, The Affiliated Traditional Chinese Medicine Hospital, Southwest Medical University, 646000 Luzhou, Sichuan, China; ^2^Department of Cardiovascular Disease, Shenzhen Traditional Chinese Medicine Hospital, 518033 Shenzhen, Guangdong, China; ^3^School of Pharmacy, Southwest Medical University, 646000 Luzhou, Sichuan, China

**Keywords:** extracellular vesicles, heart failure, functional heterogeneity, biomarkers, engineering treatment

## Abstract

Heart failure (HF) represents a class of cardiovascular diseases that poses a serious threat to global health. Although current pharmacological and device-based therapies have exhibited some progress, significant challenges remain, including suboptimal treatment responses and the inability to effectively halt disease progression. Extracellular vesicles (EVs) are nanoscale membranous particles actively secreted by cells, which are capable of transporting bioactive molecules such as nucleic acids and proteins to mediate intercellular communication. Owing to the broad cellular origins and excellent biocompatibility of EVs, these particles offer extensive therapeutic potential. This review systematically elaborates on the key aspects of EVs, including the core molecular composition of these particles, as well as the biogenesis pathways and functional regulatory mechanisms involved. We further dissect the functional heterogeneity of EVs derived from cardiomyocytes, cardiac fibroblasts, endothelial cells, and immune cells in HF, highlighting the dual roles of EVs in either promoting or counteracting disease progression via cargo-dependent mechanisms. Additionally, we explore the translational applications of EVs in the diagnosis and treatment of HF, covering EV isolation, characterization, and scalable production strategies. The potential use of EVs as biomarkers, as well as the precision engineering of EVs for targeted clinical therapy, are also critically discussed.

## 1. Introduction

Heart failure (HF) is experiencing a rising global prevalence and incidence, presenting a pressing public health challenge in cardiovascular medicine that requires urgent attention. While current standard therapies including neurohormonal antagonists and cardiac resynchronization therapy, have improved outcomes in selecting patient populations, the complex pathophysiology and significant interpatient variability of HF continue to pose major clinical challenges [1, 2]. Many patients continue to exhibit inadequate treatment responses and progressive disease deterioration, highlighting the critical need to better understand fundamental disease mechanisms and develop novel therapeutic approaches. Extracellular vesicles (EVs) are nanoscale membrane-bound particles actively secreted by cells that facilitate intercellular communication by transporting bioactive cargo including nucleic acids (miRNAs, mRNAs, lncRNAs), proteins (cytokines, enzymes), lipids and metabolites [3]. In HF, various cardiac cell types including cardiomyocytes, fibroblasts, endothelial cells and immune cells release distinct EVs populations that exert bidirectional regulatory effects on disease progression through modulation of key pathological processes such as myocardial fibrosis, inflammatory response, angiogenesis and apoptosis [4, 5, 6, 7, 8, 9].

Emerging research has demonstrated the dual diagnostic and therapeutic potential of extracellular vesicles in heart failure management [10]. Holding promise as natural or engineered therapeutic vehicles for targeted drug delivery and precise intervention. This review systematically outlines the biogenesis, molecular composition, and functional mechanisms of EVs, with a focus on the functional heterogeneity of EVs derived from different cell types in HF. Furthermore, it discusses potential clinical translational pathways of EVs in HF diagnosis and treatment, providing a theoretical foundation for advancing mechanistic research and developing novel therapeutic approaches.

## 2. Biogenesis, Composition, and Functional Regulation of Extracellular Vesicles

**Fig. 1.**
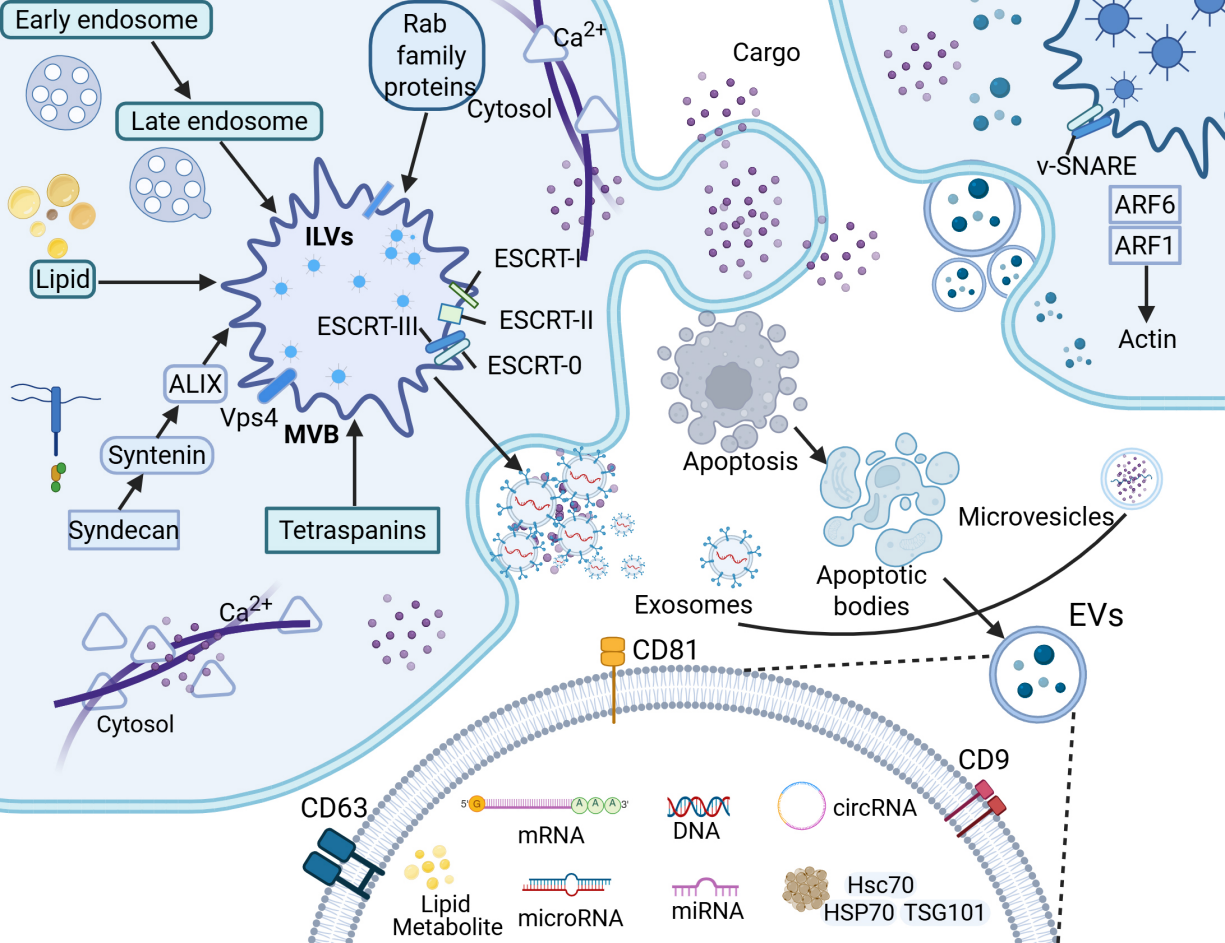
**Biogenesis, molecular composition, and regulatory mechanisms of EVs**. EVs are membrane-bound nanoparticles secreted by cells, mainly categorized into exosomes, microvesicles (MVs), and apoptotic bodies according to their distinct biogenesis pathways. Specifically, exosomes form through the endosomal system, evolving from early endosomes to multivesicular bodies; MVs are generated by direct budding from the plasma membrane; and apoptotic bodies are produced in the late stages of apoptosis. Additionally, EVs can be transported between cells and carry core biomolecular components such as proteins, lipids, and nucleic acids. EVs, extracellular vesicles; ILVs, intraluminal vesicles; ESCRT, endosomal sorting complex required for transport; ALIX, ALG-2 interacting protein X; Vps4, vacuolar protein sorting 4; MVB, multivesicular body; v-SNARE, vesicle-soluble N-ethylmaleimide-sensitive factor attachment protein receptor; HSP70, heat shock protein 70; Hsc70, heat shock cognate 70; TSG, tumor susceptibility gene; CD9, cluster of differentiation 9; CD81, cluster of differentiation 81; CD63, cluster of differentiation 63; ARF1, ADP-ribosylation factor 1; ARF6, ADP-ribosylation factor 6. Created in BioRender (https://www.biorender.com/).

The biological processes of EVs are shown in Fig. 1. The upper panel highlights the core molecular composition of EVs, clearly depicting their cargo components, including membrane proteins, functional proteins, and nucleic acids. This visualization underscores their role as natural biomolecular carriers and provides a structural basis for understanding the classification criteria, molecular profiles, and functional diversity of EVs. The lower panel delineates the biogenesis and release pathways of EVs, encompassing the formation and regulatory mechanisms of exosomes, microvesicles (MVs) and apoptotic bodies. Key regulatory factors and stimuli are indicated to elucidate the dynamic regulation of EVs secretion.

### 2.1 Biological Pathways and Classification of Extracellular Vesicles

EVs are widely distributed and derived from diverse cellular sources [11]. Their biogenesis pathways exhibit remarkable heterogeneity and specificity, with distinct EV subtypes displaying unique biological characteristics attributable to their formation mechanisms.

Exosomes biogenesis initiates through the endosomal pathway, where plasma membrane invagination forms early endosomes that subsequently mature into late endosomes [12, 13]. The limiting membrane of late endosomes undergoes inward budding to encapsulate cytoplasmic components, generating intraluminal vesicles (ILVs) within multivesicular bodies (MVBs) [3]. In contrast, MVs are generated via direct plasma membrane outward budding, which packages cytosolic biomolecules before detaching from the parental cell membrane. MVs typically range from 100 nm to 1 µm in diameter [14]. Notably, exosomes and MVs share overlapping molecular compositions, including adhesion molecules, membrane receptors, tissue factors, cytoskeletal proteins, chemokines, enzymes, cytokines, and nucleic acids [15]. Apoptotic bodies, a distinct EV subtype, are produced exclusively during programmed cell death or apoptosis. With diameters of 1–5 µm, they are significantly larger than other EVs constitutively released by viable cells [14, 16].

### 2.2 Core Molecular Composition of Extracellular Vesicles

The core molecular composition of EVs is complex and highly diversified, mainly including membrane proteins, nucleic acids, functional proteins, lipids and metabolites, etc. These molecules jointly endow EVs with biological functions and play a key role in intercellular communication [17, 18, 19, 20]. Membrane proteins are not only the surface markers of EVs such as Cluster of Differentiation 9 (CD9), Cluster of Differentiation 63 (CD63) and Cluster of Differentiation 81 (CD81), but also actively participate in the generation of EVs, screening of contents, targeted recognition and cellular uptake [21]. EVs carry nucleic acids including miRNAs, mRNA, circRNAs, and mitochondrial Deoxyribonucleic Acid (DNA). These nucleic acid molecules can be transferred to recipient cells to regulate their gene expression and functional states [22]. Additionally, circRNAs, due to their circular structure that resists degradation, exhibit more enduring effects in intercellular regulation [23]. Functional proteins such as heat shock proteins (e.g., Heat Shock Cognate 70 [Hsc70], Heat Shock Protein 70 [HSP70]) and endosomal sorting complex required for transport (ESCRT)-related proteins such as *tumor susceptibility gene 101* are not only involved in the formation of MVBs, intracellular vesicles and exosomes, but also may affect the stress response and immune function of receptor cells [24]. The composition of lipids (such as cholesterol, sphingolipid and phosphatidic acid) not only maintains the structural integrity of EVs, but also participates in signal transduction and membrane fusion processes, which affect the interaction ability between EVs and target cells [25]. Recent studies have shown that EVs also carry a range of metabolites such as fatty acids, amino acids and ketones, which can reflect the metabolic status of cells and may mediate distal effects such as metabolic reprogramming, with potential pathological significance in metabolic stress diseases such as heart failure [26].

### 2.3 Function and Regulatory Mechanism of Extracellular Vesicles

The functional implementation of EVs relies on a cascade of regulatory processes from biogenesis to targeted action. During the intracellular phase, the biosynthesis of ILVs is governed by sophisticated mechanisms, primarily involved both ESCRT-dependent and ESCRT-independent pathways. The ESCRT-dependent pathway comprises four distinct complexes (ESCRT-0, -I, -II, and -III) and accessory proteins (e.g., Vacuolar Protein Sorting 4 [Vps4], ALG-2 Interacting Protein X [ALIX]), each performing specialized regulatory functions [27, 28]. In contrast, the ESCRT-independent pathway employs alternative mechanisms such as the Syndecan-Syntenin-ALIX axis, lipid rafts, tetraspanins, and Rab family guanosine triphosphatases (GTPases), collectively contributing to ILVs formation and cargo sorting, thereby demonstrating the complexity of exosome biogenesis [29]. Microvesicle generation similarly involves coordinated action of multiple molecules. Luminal lipidated proteins (e.g., myristoylated and palmitoylated proteins) may facilitate membrane curvature, while certain ESCRT subunits (I/II/III) participate in microvesicle assembly and budding. Additionally, ceramide, which is produced through acid sphingomyelinase activation contributes to plasma membrane-derived release [29]. Apoptotic bodies emerge during late-stage programmed cell death, regulated by caspase-mediated cleavage and subsequent Rho-associated protein kinase activation [16, 30]. These factors coordinate cytoskeletal reorganization and membrane dynamics to control apoptotic body formation. Upon entering circulation, EVs undergo microenvironmental modifications including membrane’s asymmetry establishment, polarization within pH gradients, localized deformation and migration, as well as surface corona formation. When reaching target cells, integrin family proteins mediate tissue-specific homing, followed by functional cargo delivery via membrane fusion or endocytic pathways, ultimately reprogramming recipient cell signaling networks [31]. This series of precisely regulated processes not only ensures EVs’ accurate participation in intercellular communication but also provides mechanistic foundations for developing EV-based therapeutic interventions.

The extracellular matrix (ECM) plays an indispensable role in the generation of EVs [32]. Through mechanical transduction signals, the stiffness and viscoelastic properties of the ECM remodel actin cytoskeletal assembly and regulate membrane raft dynamics, thereby directly influencing vesicle formation efficiency [33, 34]. Additionally, EVs themselves participate in multiple ECM regulatory functions including matrix degradation, protein cross-linking, and calcification [35]. Notably, the ECM also modulates vesicle cargo loading by balancing non-coding RNA expression within cells [36]. For instance, after myocardial infarction, specific miRNAs carried by exosomes released by endocardial and epicardial cells require ECM-mediated intercellular communication to achieve targeted delivery [34].

## 3. Functional Heterogeneity of EVs From Different Cardiac Cell Sources in HF

**Fig. 2.**
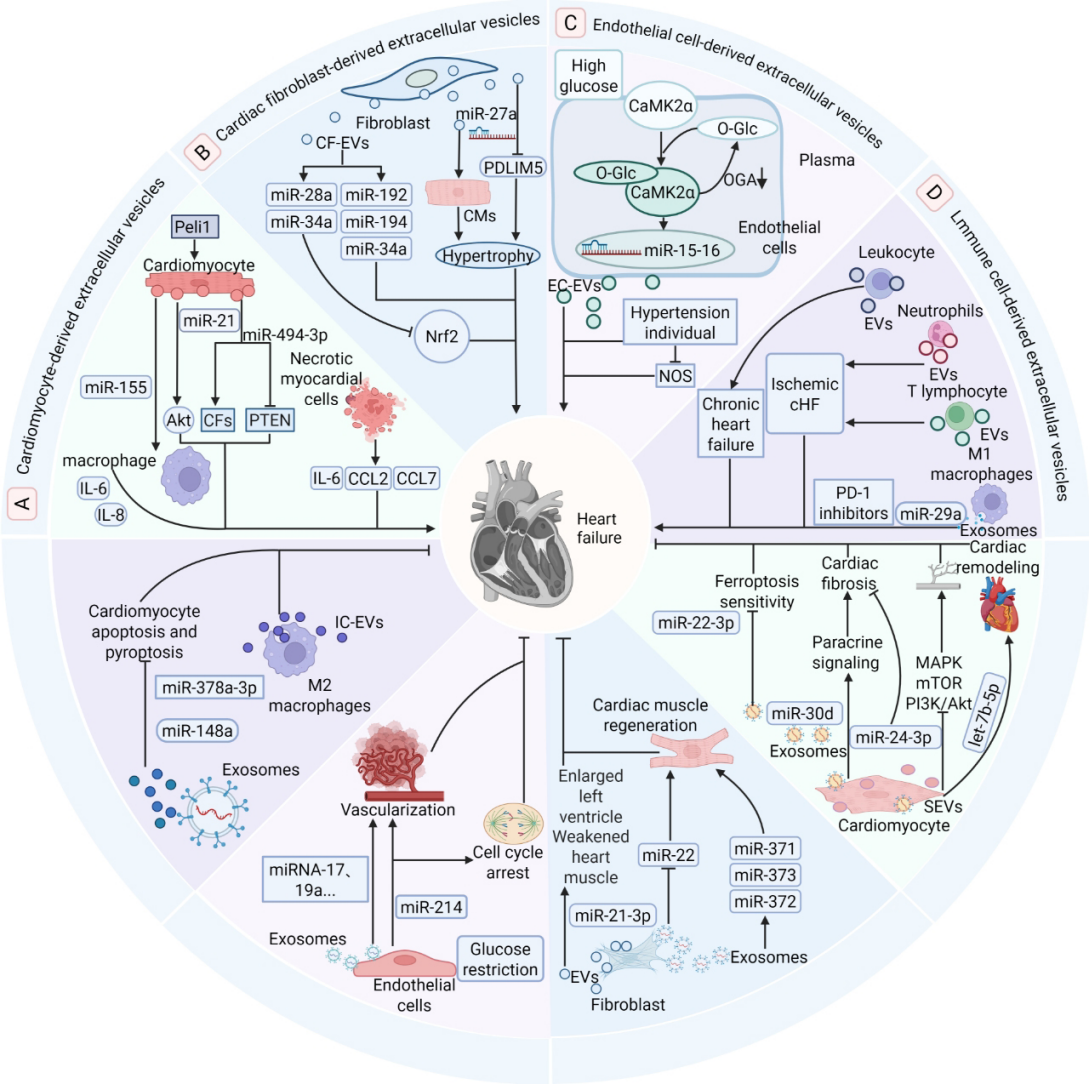
**Bipartite functional roles of EVs from cardiomyocyte, fibroblast, endothelial, and immune cells in heart failure**. EVs derived from specific cell types exert critical yet opposing functions in the progression of heart failure. This diagram summarizes the pathogenic and protective roles of vesicles released from four distinct cell sources, which are denoted by different background colors; areas sharing the same color represent the dual functions of vesicles originating from an identical cell type. (A–D) The upper section illustrates the pathological mechanisms through which these vesicles promote the initiation and progression of heart failure by targeting downstream signaling pathways. In contrast, the lower section demonstrates the protective effects mediated by extracellular vesicles, which attenuate heart failure through diverse molecular mechanisms. Akt, protein kinase B; CaMK2α, calcium/calmodulin-dependent protein kinase IIα; CF, cardiac fibroblast; CCL7, chemokine ligand 7; CMs, cardiomyocytes; EC, endothelial cell; cHF, chronic heart failure; MAPK, mitogen-activated protein kinase; mTOR, mammalian target of rapamycin; NOS, nitric oxide synthase; Nrf2, nuclear factor erythroid 2-related factor 2; O-Glc, O-GlcNAcylation; OGA, O-GlcNAcase; PD-1, programmed cell death protein 1; PI3K/Akt, phosphatidylinositol 3-kinase/protein kinase B; PTEN, phosphatase and tensin homolog; SEVs, small extracellular vesicles. Created in BioRender (https://www.biorender.com/).

EVs derived from distinct cardiac cell populations exhibit functional heterogeneity in the pathogenesis of heart failure, with their cargo molecules participating in either pro-failure or cardioprotective effects through complex regulatory networks, collectively constituting a multidimensional modulation system in heart failure progression. As illustrated in Fig. 2A–D, the specific functions and molecular mechanisms of cardiomyocyte-derived, cardiac fibroblast-derived, endothelial cell-derived, and immune cell-derived EVs in heart failure are presented systematically. The upper section demonstrates the pro-heart failure functions and molecular mechanisms of EVs derived from cardiomyocytes, cardiac fibroblasts, endothelial cells, and immune cells, clarifying how they accelerate HF pathogenesis by regulating downstream signaling pathways. The lower section correspondingly presents the anti-heart failure effects and related mechanisms of these four cell-derived EVs, revealing their crucial role in delaying disease progression.

### 3.1 Cardiomyocyte-Derived Extracellular Vesicles (CM-EVs)

CM-EVs exhibit dual regulatory roles in HF through their cargo of diverse miRNAs, proteins, and cytokines. In their pathological capacity, CM-EVs from ischemic myocardium are internalized by infiltrating monocytes, triggering increased production of pro-inflammatory cytokines and chemokines that exacerbate HF. Xia and Wang [37] demonstrated that stress-induced CM-EVs enriched with *miR-21* promote post-ischemic cardiac fibrosis via the activation of protein kinase B (AKT) signaling pathway. Moreover, EVs from necrotic cardiomyocytes enhance inflammatory responses by stimulating interleukin-6 (IL-6), CC motif chemokine ligand 2 (CCL2), and CC motif chemokine ligand 7 (CCL7) release upon monocyte uptake [38]. Tang *et al*. [39] revealed that Peli1-induced CM-EVs carrying *miR-494-3p* activate cardiac fibroblasts through phosphatase and tensin homolog (PTEN) suppression and subsequent hyperphosphorylation of Akt, Mothers Against Decapentaplegic Homolog 2/3 (SMAD2/3), and Extracellular Signal-regulated Kinase (ERK), thereby accelerating fibrosis. Additional studies showed that hypertrophic CM-EVs transfer *miR-155* to macrophages, inducing pro-inflammatory cytokine (IL-6/IL-8) release [40]. Conversely, cardioprotective CM-EVs deliver beneficial effects: *miR-30d*-containing vesicles attenuate cardiac fibrosis, apoptosis, and hypertrophy to improve the function [41]. Senesi *et al*. [42] reported that induced CM-EVs carrying *miR-24-3p* suppress cardiac fibrosis, mitigating HF progression. Healthy adult CM-EVs multi-targetedly inhibit pro-fibrotic pathways Mitogen-activated Protein Kinase (MAPK), mammalian Target of Rapamycin (mTOR), Janus kinase/signal transducer and Activator of Transcription (JAK/STAT), Transforming Growth Factor Beta (TGFβ), Phosphatidylinositol 3-kinase (PI3K)/Akt, reversing fibroblast activation, reducing extracellular matrix deposition, and promoting angiogenesis [23]. Zhang *et al*. [43] identified *let-7b-5p* as mediating cardiac remodeling through small EV-mediated delivery. Emerging evidence showed that CM-exosomal *miR-22-3p* targets acyl-coA synthetase long-chain family member 4 to inhibit ferroptosis susceptibility in ischemic HF [31]. These findings underscore CM-EVs’ functional dichotomy in HF pathogenesis and therapy. Their cargo-specific heterogeneity and molecular mechanisms warrant further exploration to develop precision interventions. Current evidence showed CM-EVs as both disease mediators and therapeutic vectors, with their net effects depending on parental cell status and pathological context.

### 3.2 Cardiac Fibroblast-Derived Extracellular Vesicles (CF-EVs)

Similar to CM-EVs, CF-EVs exhibit dual functional roles in HF. CF-EVs contribute to HF progression by delivering pathological cargo to cardiomyocytes. Qiao *et al*. [44] demonstrated that CF-EVs transport miRNAs that activate hypertrophic signaling pathways, promoting cardiomyocyte hypertrophy. Under oxidative stress, *miR-27a* enriched fibroblast-derived exosomes suppress PDZ and LIM domain 5 (PDLIM5) expression, exacerbating cardiac hypertrophy in a myocardial infarction induced chronic HF model [45]. In patients with HF post-acute myocardial infarction, circulating exosomal levels of *miR-192*, *miR-194*, and *miR-34a* are significantly elevated, with *miR-194* and *miR-34a* correlating positively with left ventricular diastolic dimensions and negative with ejection fraction [46]. Additionally, Tian *et al*. [47] reported that stress-responsive miRNAs (e.g., *miR-28a*, *miR-34a*) are upregulated in cardiac fibroblasts, packaged into EVs, and secreted to suppress nuclear factor erythroid 2-related factor 2 (Nrf2) translation, directly impairing cardiomyocyte function. Conversely, EVs may exert beneficial effects under certain conditions. Studies have shown that exosomes derived from induced pluripotent stem cells differentiated cardiac fibroblasts from HF patients exhibit reduced *miR-22* but elevated *miR-371/372/373* expression, suggesting a role in promoting myocardial repair [48]. Bang *et al*. [49] identified that CF-EVs carrying *miR-21-3p* mediate cardiomyocyte hypertrophy, yet this mechanism may also be harnessed for therapeutic regeneration. These findings highlight CF-EVs as potential targets for modulating cardiac remodeling and fibrosis in HF.

### 3.3 Endothelial Cell-Derived Extracellular Vesicles (EC-EVs)

EC-EVs, as a key cellular component maintaining cardiovascular homeostasis, demonstrate dual regulatory functions in the pathogenesis of HF, working synergistically with cardiomyocyte- and cardiac fibroblast-derived EVs to participate in the complex pathological network of HF. Suades *et al*. [50] found that circulating extracellular vesicles (cEVs) derived from endothelial cells are mainly phosphatidylserine circulating extracellular vesicles (PS^–^-cEVs) and significantly elevated in chronic HF patients, though their precise mechanistic role in HF remains unclear. Regarding HF progression promotion, studies have shown that high glucose-induced CaMK2a/O-GlcNAcylation positive feedback loop in endothelial cells leads to sustained calcium/calmodulin-dependent protein kinase IIα (CaMK2a) activation, subsequently generating plasma small EVs (sEVs) [51]. Arterial endothelial cells serve as the primary source of *miR-15-16* in these sEVs, which exert persistent detrimental effects on cardiomyocytes and induce cardiac dysfunction in healthy animals. Conversely, in HF antagonism, research demonstrates that glucose-deprived H9C2 rat cardiomyocytes can modulate endothelial cell-released exosomes overexpressing various miRNAs (*miR-17*, *miR-19a*, etc.), thereby promoting angiogenesis [52]. Furthermore, under physiological conditions, EC-EVs influenced by cardiomyocytes exhibit a high proportion of vesicular transport-related proteins. Van Balkom *et al*. [53] discovered that endothelial cell EC-derived exosomes enriched with *miR-214* promote angiogenesis and prevent cell cycle arrest in recipient ECs, potentially exerting protective effects by improving myocardial blood supply. Another study revealed that circulating EC-EVs from hypertensive individuals may potentially increase HF risk by upregulating proteins associated with cardiac hypertrophy and fibrosis while downregulating nitric oxide synthase (NOS) expression [54]. Comprehensive elucidation of EC-EVs functional heterogeneity and specific regulatory mechanisms will provide novel insights and experimental basis for precision-targeted therapy of heart failure.

### 3.4 Immune Cell-Derived Extracellular Vesicles (IC-EVs)

As crucial components of the cardiac microenvironment, IC-EVs exhibit complex dual regulatory roles in HF pathogenesis, working in concert with EVs from cardiomyocytes, cardiac fibroblasts, and endothelial cells to modulate HF progression. Experimental studies have demonstrated elevated levels of leukocyte-derived EVs in chronic HF patients compared to controls, with lymphocyte- and neutrophil-derived EVs being specifically detected in chronic heart failure (cHF). Ischemic cHF patients show significantly increased EVs production from T lymphocytes and neutrophils, with immune cell-derived EVs correlating with New York Heart Association (NYHA) functional class and being similarly activated in both heart failure with preserved ejection fraction (HFpEF) and heart failure with reduced ejection fraction (HFrEF) patients [5]. Gąsecka *et al*. [55] revealed that elevated leukocyte EVs concentrations are associated with a 4.7-fold increased risk of systolic dysfunction progression in heart failure with mid-range ejection fraction (HFmrEF) patients, indicating the pro-HF effects of IC-EVs across cHF subtypes. Additional studies demonstrated that M1 macrophage-derived exosomes can induce cardiomyocyte pyroptosis via *miR-29a* [56], while programmed death 1 (PD-1) inhibitor-treated macrophage exosomes promote cardiac senescence-related damage through the *microRNA-34a-5p*/PNUTS signaling pathway [57]. Conversely, M2 macrophage-derived EVs exhibit cardioprotective properties, with studies showing that exosomes carrying *miR-148a* and *miR-378a-3p* can reduce cardiomyocyte apoptosis and pyroptosis post-cardiac injury [58, 59]. These findings position IC-EVs as key mediators in HF’s complex pathological network and provide a theoretical foundation for developing immune-targeted precision therapies.

## 4. The Transformation Pathway of EVs in the Diagnosis and Treatment of HF

**Fig. 3.**
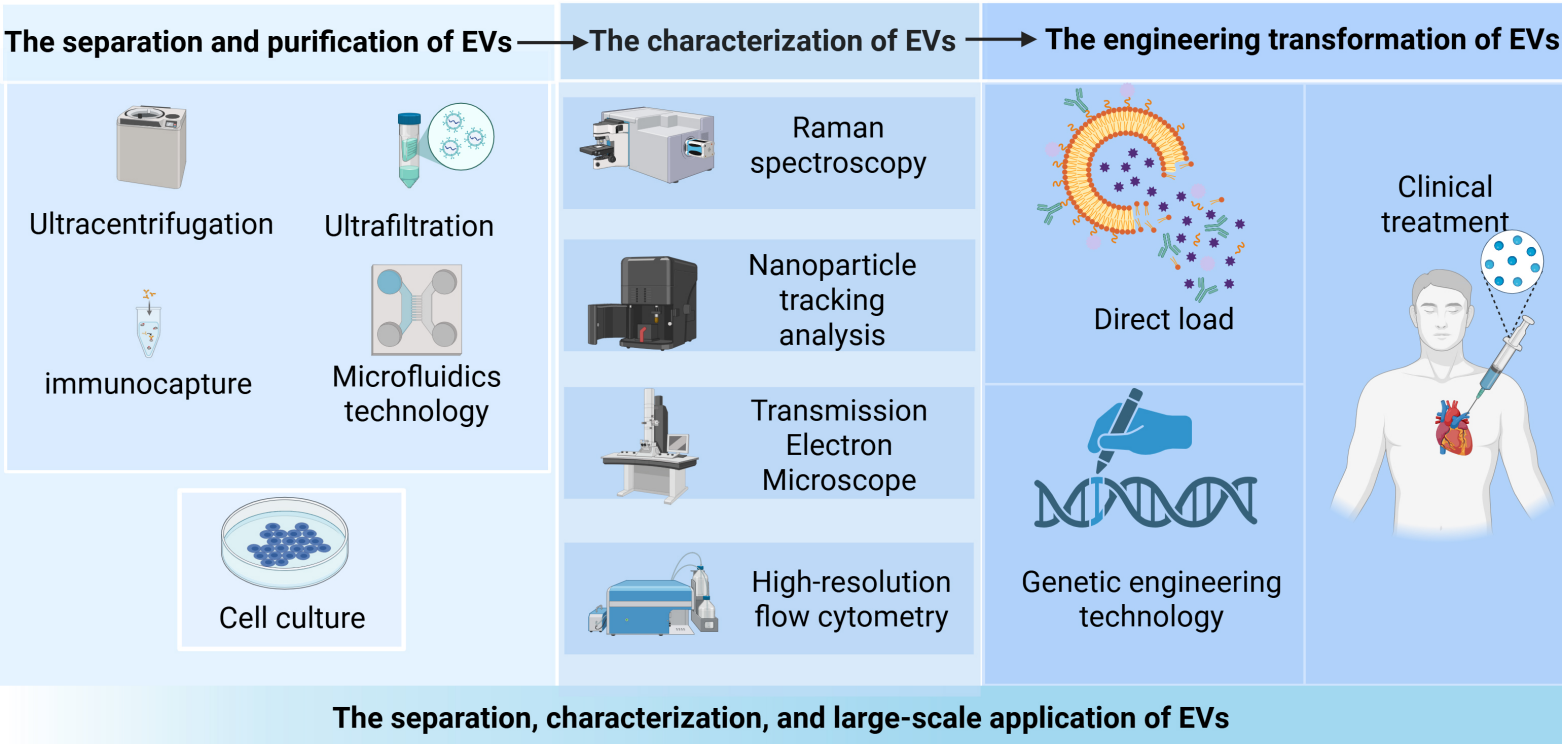
**The separation, characterization, and large-scale application of EVs**. The isolation and purification of EVs are first achieved through ultracentrifugation and ultrafiltration techniques, followed by characteristic analysis using Raman spectroscopy and transmission electron microscopy. Through direct loading or genetic engineering approaches for subsequent improvements, these vesicles show promise as an effective therapeutic approach for heart failure treatment, paving the way for their clinical application in the future. Created in BioRender (https://www.biorender.com/).

The transformation of EVs from basic mechanism research to clinical diagnosis and treatment applications is a multi-link collaborative system engineering, covering key steps such as separation and purification, characterization technology, engineering transformation, and large-scale application, ultimately achieving the goal of clinical treatment. The key pathways involved in the production of extracellular vesicles are shown in Fig. 3.

### 4.1 Separation, Characterization, and Large-Scale Application of EVs

EVs are present in various biological fluids including blood, milk, and saliva, where they play important roles in long-distance cellular communication [60]. Although conventional wisdom suggests that fresh samples typically yield higher quantities of EVs, this is often difficult to achieve in practice. The isolation of EVs presents significant challenges due to their nanoscale size characteristics and frequent co-isolation with contaminants such as cellular debris and lipoproteins. Moreover, different isolation methods can substantially influence downstream analyses of EV cargo composition and physicochemical properties [61]. Current isolation techniques primarily include ultracentrifugation, ultrafiltration (also called microfiltration), precipitation, immunoaffinity capture, microfluidic technologies, and commercial kits [62, 63, 64, 65]. In terms of characterization, EVs analysis are advancing toward multidimensional precision profiling. EVs are typically characterized by their size, concentration, presence of protein markers, protein content, and other components. The main single-particle EVs analysis technologies currently available include nanoparticle tracking analysis, microscopy techniques (electron microscopy, cryo-electron microscopy, atomic force microscopy, and high-resolution microscopy), resistive pulse sensing, high-resolution flow cytometry, and Raman spectroscopy [66].

Current medical capabilities have yet to achieve large-scale clinical application of EVs, primarily due to technical challenges in EV isolation and production, as well as compositional variability [67, 68]. While production yields can be enhanced through various approaches including culture condition optimization (moderate pH adjustment, modulation of calcium ion concentration, glucose content, and oxygen levels), application of physical stimuli (temperature, light, or electrical pulses), or genetic modification to regulate EVs biogenesis-related proteins (e.g., Rab proteins and phospholipase D), the impacts of these interventions on EVs cargo composition, functionality, and stability require further investigation [69]. Realizing scalable applications necessitates comprehensive system-wide optimization: employing microcarriers with chemically defined media in cell culture to increase yield while preventing contamination; integrating size-exclusion chromatography with immunoaffinity capture during purification for efficient removal of lipoproteins and cellular debris; and establishing modular bioreactors with integrated “culture-purification-quality control” workflows to ensure EVs functional stability, thereby laying the foundation for large-scale production. This holistic approach not only addresses current technical limitations but also provides a clear pathway toward standardized, high-quality EVs manufacturing for clinical applications.

### 4.2 Extracellular Vesicles as Biomarkers for HF

Currently, traditional biomarkers such as brain natriuretic peptide and N-terminal pro-brain natriuretic peptide are widely used in clinical diagnosis of HF, yet their limitations including insufficient sensitivity for early diagnosis and significant interference from non-cardiac factors remain unresolved [2, 70, 71]. Consequently, there is growing interest in identifying biomarkers with superior sensitivity and specificity to optimize HF clinical management. As key mediators of intercellular communication, EVs carry nucleic acid molecules (e.g., miRNAs, lncRNAs) which directly reflect the physiological or pathological status of their parent cells, emerging as a promising research focus for HF diagnosis, prognosis evaluation, and disease monitoring [72]. Wu *et al*. [73] demonstrated upregulated expression of EV-associated *miR-92b-5p* in serum from acute HF patients with dilated cardiomyopathy, suggesting its potential as a diagnostic biomarker for AHF secondary to this condition. Studies have confirmed significantly elevated levels of exosomal *miR-192-5p* and *miR-320a* in patients with acute decompensated HF compared to healthy controls. These miRNAs show positive correlations with patient age and cardiac chamber dimensions, exhibiting negative correlations with left ventricular ejection fraction and fractional shortening, which collectively indicate their capacity to reflect HF progression through associations with cardiac functional parameters [74, 75]. Notably, EV-encapsulated *miR-126* and *miR-199a* demonstrate direct clinical relevance to cardiovascular outcomes, unlike their free circulating counterparts which show no clear association with HF-related events, highlighting the unique prognostic value of EV-packaged miRNAs for precise patient stratification and personalized intervention [76]. Furthermore, EVs characteristics including concentration, size distribution, and zeta potential, show diagnostic and prognostic potential in HF. Specifically, EVs concentration correlates strongly with overall survival in HF patients, where lower concentrations predict poorer outcomes, suggesting the utility of EVs profiling for prognostic assessment [77].

### 4.3 Precise Design and Clinical Treatment of Engineered EVs

Beyond their utility as HF biomarkers, EVs possess inherent therapeutic advantages including excellent biocompatibility, low immunogenicity, and the ability to cross biological barriers, making them ideal nanocarriers for HF treatment. Through multifunctional design strategies and engineered modifications, EVs can be further optimized to enhance targeting efficiency, payload capacity, and therapeutic efficacy, thereby providing critical support for reversing myocardial remodeling and improving clinical outcomes in HF.

The conjugation of ischemic myocardium-targeting peptides or cardiac homing peptides to EVs surfaces enhances their specific targeting capability toward ischemic or injured myocardium, enabling efficient delivery of therapeutic molecules such as miRNAs to reduce apoptosis and restore cardiac function. Studies demonstrate that hypoxia-preconditioned BM-MSCs-derived EVs enriched with *miR-125b-5p*, when coupled with ischemic myocardium-targeting peptides and administered intravenously, these achieve highly specific delivery of cardioprotective miRNAs to the ischemic myocardium [78, 79, 80]. Similarly, cardiac homing peptide-modified stem cell-derived EVs facilitate targeted delivery of *miR-21* gene therapy, improving cardiac functional recovery post myocardial infarction while attenuating subsequent heart failure development [81]. Mentkowski and Lang [82] experimentally verified that fusion of cardiomyocyte-specific binding peptides (CMPs) with the exosomal transmembrane protein LAMP2b significantly enhanced cardiomyocyte-specific uptake *in vitro*. Following intramyocardial injection in *vivo*, CMP-modified exosomes markedly reduced cardiomyocyte apoptosis, suggesting their therapeutic potential for heart failure treatment [52].

Functionalized EVs loaded with therapeutic molecules (e.g., antifibrotic drugs, pro-angiogenic factors, or nucleic acids) enable precise targeting of key pathways in HF progression. Ma *et al*. [83] demonstrated that exosomes derived from *miR-132* mimic-treated BM-MSCs enhance angiogenesis and improve cardiac function in MI mice through targeted suppression of RASA1. Similarly, engineered EVs from cardiosphere-derived cells, which are naturally enriched with cardioprotective nucleic acids (*miR-4488*, *miR-92a*), attenuate atrial fibrosis-related conduction abnormalities and electrical dyssynchrony when administered intravenously in HFpEF rats. This effect is mediated through coordinated modulation of pro-inflammatory, pro-fibrotic, and anti-angiogenic pathways, resulting in significantly reduced atrial fibrillation incidence [84].

EVs derived from genetically modified or specific stem cell sources, which inherently carry therapeutic molecules with enhanced bioactivity, have been demonstrated to play significant roles in promoting angiogenesis and improving cardiac function. Studies have shown that adipose-derived mesenchymal stem cell exosomes (ADSC-Exos) can mitigate cardiac injury and enhance functional recovery in myocardial infarction (MI) mice through the *miR-205* signaling pathway, suggesting the considerable therapeutic potential of *miR-205*-enriched ADSC-Exos for treating MI-induced heart failure [85, 86]. Comparative investigations revealed that exosomes isolated from Akt-modified human umbilical cord-derived mesenchymal stem cells induced more pronounced improvements in both angiogenesis and cardiac function than their unmodified counterparts, highlighting the superior therapeutic efficacy of genetically engineered EVs [87].

However, the clinical translation of engineered EVs remains constrained by multiple practical challenges, necessitating a more objective and rigorous perspective to evaluate their development prospects. In terms of core therapeutic efficacy, insufficient targeting specificity continues to be an unbroken bottleneck, while surface-anchored targeting peptides can enhance tropism toward damaged myocardium, complex circulatory dynamics and physiological microenvironments *in vivo* often lead to ligand degradation, shedding, or nonspecific binding to non-target organs. Some modified EVs still exhibit high off-target rates, which not only reduces drug concentration at lesion sites but may also increase potential risks in non-target tissues. Regarding industrial implementation, exorbitant production costs severely limit large-scale applications: The engineering processes involving ligand modification and precise therapeutic molecule loading require highly sophisticated equipment and technical precision. Moreover, the low efficiency and limited yield of high-purity engineered EVs result in per-dose treatment costs far exceeding conventional drugs, making them unsuitable for clinical batch production. On regulatory and safety fronts, the absence of clear approval standards and unknown long-term toxicity pose dual obstacles [88]. The clinical translation of engineered EVs therapies is hindered by the lack of standardization in classification, quality control, and regulatory approval. Additionally, critical long-term safety data remain scarce. Potential issues such as genomic integration risks associated with gene-edited EVs, immune memory reactions triggered by heterologous modified ligands, and toxic side effects from prolonged molecular accumulation in patients—most existing research focuses on short-term animal experiments rather than long-term clinical follow-up evidence. In addition, there are few clinical cases at present, and no large-scale, multi-center randomized controlled trial has been conducted to confirm its efficacy and safety in HF patients with different subtypes and stages.

Despite current challenges including scalable production, long-term safety validation, and limited clinical application, engineered EVs are poised to emerge as a novel therapeutic modality for reversing myocardial remodeling and improving HF prognosis as design technologies mature, thereby ushering in a new era of precision medicine for heart failure.

## 5. Discussion

In the research field of EVs for heart failure diagnosis and treatment, several key directions demonstrate significant scientific value and translational potential: (1) In-depth elucidation of the roles of EV-carried non-coding RNAs and proteins in regulating core myocardial repair pathways not only helpfully clarifies the molecular mechanisms of EV-mediated cardiac regeneration, but also establishes a theoretical foundation for identifying precise therapeutic targets. (2) EVs exhibit dual clinical potential as both disease-specific biomarkers and therapeutic vectors. As natural carriers of cellular cargo molecules, EVs possess the advantages of noninvasive sampling and dynamic monitoring capabilities, providing novel biomarker sources for clinical diagnosis. Meanwhile, engineered strategies are progressively overcoming *in vivo* delivery bottlenecks, making EVs promising as future clinical therapeutic tools. (3) Multidisciplinary integration provides strong driving force for innovative EVs research development. By combining molecular biology, materials science, clinical medicine and other interdisciplinary technologies, targeted modifications can enhance EVs targeting specificity, sustained-release properties and therapeutic efficacy, effectively promoting the translation from basic mechanistic research to diagnostic and therapeutic applications, and accelerating the process of bringing EVs from laboratory to clinic.

Despite the promising application prospects of EVs, current clinical research still faces several limitations: (1) The biological functions of EVs derived from different cellular sources are intricately influenced by cargo molecules, microenvironmental factors, and cellular states, with their precise regulatory networks remaining incompletely elucidated. Furthermore, the lack of standardized classification criteria correlating EV subtypes with specific functions has constrained both fundamental understanding of EVs biology and subsequent translational applications. (2) Engineered EVs still exhibit limitations in targeting precision. Although surface modifications can confer some targeting capability, current systems demonstrate suboptimal specificity and enrichment efficiency for particular tissues, cells, or pathological lesions. The prevalent issue of nonspecific distribution restricts their efficacy as delivery vehicles or therapeutic tools, while also posing challenges for clinical translation regarding safety and effectiveness. (3) Significant technical and standardization barriers persist in EVs clinical translation. Existing isolation and purification technologies remain inadequate for large-scale clinical applications, and the long-term safety as well as mass production stability of engineered EVs await comprehensive validation through large-scale clinical trials-factors that fundamentally limit their transition from bench to bedside. Additionally, existing studies mostly rely on small sample animal experiments or *in vitro* models, and most of the research is limited to the pre-clinical stage. Large-scale clinical trial data are still blank.

To address these challenges, focused efforts should be directed toward several key areas to enhance the translational potential of EVs: (1) Future studies should integrate cutting-edge technologies such as single-cell sequencing and proteomics to elucidate the cargo-sorting mechanisms of EVs and their precise intercellular communication networks, which will provide more accurate therapeutic targets for intervention and establish a theoretical foundation for translating basic EV research into clinical applications. (2) The advancement of efficient and cost-effective separation technologies and scalable production processes for engineered EVs is crucial for establishing industry standards, aligning regulatory reviews, and ultimately accelerating clinical translation refering to the latest EV research and application guidelines released by the International Society for Extracellular Vesicles (ISEV) for reference. (3) Exploration of EV-based multi-omics biomarker panels for constructing early diagnostic models of heart failure, combined with investigation of synergistic applications between engineered EVs and existing therapeutic modalities, which should be prioritized. Concurrently, large-scale clinical trials are needed to systematically validate long-term efficacy and safety, thereby providing high-level evidence to support standardized clinical implementation of EVs in heart failure management.

## 6. Conclusion

EVs have emerged as pivotal mediators of intercellular communication, demonstrating remarkable potential in both the pathological mechanisms and clinical management of heart failure. This review systematically synthesizes current knowledge regarding EV-mediated mechanisms in HF progression, with particular emphasis on the functional heterogeneity and dual roles exhibited by EVs derived from distinct cardiac cell populations. While EVs may exacerbate HF through pro-fibrotic and pro-inflammatory processes, their diagnostic value as biomarkers and therapeutic potential following engineering modifications are equally noteworthy. By comprehensively examining EVs’ biogenesis, functional regulation, and translational pathways in HF diagnosis and treatment, we establish a theoretical framework for their application as novel biomarkers, therapeutic vectors, and intervention targets. These insights are expected to accelerate the clinical translation of EV-based approaches for HF and other cardiovascular diseases, thereby advancing the field of precision medicine.

## Abbreviations

AKT, Protein Kinase B; ALIX, ALG-2 Interacting Protein X; ADSC-Exos, Adipose-derived Mesenchymal Stem Cell Exosomes; BM-MSCs, Bone Marrow Mesenchymal Stem Cells; CD9, Cluster of Differentiation 9; CD63, Cluster of Differentiation 63; CD81, Cluster of Differentiation 81; CMP, Cardiomyocyte-specific Binding Peptide; CM-EVs, Cardiomyocyte-derived Extracellular Vesicles; CF-EVs, Cardiac Fibroblast-derived Extracellular Vesicles; CaMK2a, Calcium/calmodulin-dependent Protein Kinase IIα; cHF, chronic Heart Failure; circRNA, circular RNA; DNA, Deoxyribonucleic Acid; EVs, Extracellular Vesicles; ERK, Extracellular Signal-regulated Kinase; ECM, Extracellular Matrix; ESCRT, Endosomal Sorting Complex Required for Transport; EC-EVs, Endothelial Cell-derived Extracellular Vesicles; GTP, Guanosine Triphosphate; HF, Heart Failure; Hsc70, Heat Shock Cognate 70; HSP70, Heat Shock Protein 70; HFpEF, Heart Failure with Preserved Ejection Fraction; HFrEF, Heart Failure with Reduced Ejection Fraction; HFmrEF, Heart Failure with mid-range Ejection Fraction; ILVs, Intraluminal Vesicles; IC-EVs, Immune Cell-derived Extracellular Vesicles; JAK/STAT, Janus kinase/signal transducer and Activator of Transcription; Lamp2b, Lysosome-associated Membrane Protein 2b; lncRNA, long non-coding RNA; MVs, Microvesicles; MVBs, Multivesicular Bodies; MAPK, Mitogen-activated Protein Kinase; mRNA, messenger RNA; miRNA, microRNA; mTOR, mammalian Target of Rapamycin; NOS, Nitric Oxide Synthase; Nrf2, Nuclear Factor Erythroid 2-related Factor 2; O-GlcNAcylation, O-linked N-acetylglucosamine Modification; PD-1, Programmed Death 1; PI3K, Phosphatidylinositol 3-kinase; PTEN, Phosphatase and Tensin Homolog; PDLIM5, PDZ and LIM domain 5; PS⁻-cEVs, Phosphatidylserine-negative Circulating Extracellular Vesicles; TGFβ, Transforming Growth Factor Beta; SMAD2/3, Mothers Against Decapentaplegic Homolog 2/3; sEVs, Small Extracellular Vesicles; Vps4, Vacuolar Protein Sorting 4.
